# Supplementary material for: Impact of parental marital status on self-harm in Chinese primary school students: the mediating role of depression and the moderating effect of classmate relationships
Source: PeerJ. 2025 Apr 4;13:e19307. doi: 10.7717/peerj.19307 (PMC11974514; doi:10.7717/peerj.19307)
Supplement: Supplemental Information 2 [file peerj-13-19307-s002.docx]

1. 学校
2. 年级：1-6
3. 学号
4. 性别：1.女 2.男
5. 年龄
6. 户籍所在地：1.城镇 2.农村
7. 您父母的关系如何？1.好 2.一般 3. 不好
8. 您父母的婚姻状态如何？1.正常婚姻状态 2.未离异但分居 3.离异
9. 您的家庭经济状况如何？ 1.好 2.一般 3.差
10. 您是否有兄弟姐妹？ 0.否 1.是
11. 您的生活主要由谁照顾？1.父母 2.除父母外的其他亲人 3.住校 4.无人
12. 您与父母的关系如何？1.好 2.一般 3.差
13. 您在学校与同学的关系如何？1.好 2.一般 3.差
14. 您觉得学习压力大吗？0.不大1.一般 2.大
15. 您上网或玩手机的频率如何？1.很少使用2.偶尔使用3.经常使用
16. 【备注：若4题性别为1.女性，则显示该题目；若否，则跳转到17题】您月经初潮的年龄 岁。（没有月经初潮请填“0”）【备注：该题目仅限填写数字，且数字不能超过其年龄（第5题）】
17. 在学校有没有人欺负您？ 1.没有 2.很少 3.多次 4.经常【备注：若本题选1.没有，则跳至19题；若否，则继续问18题】
18. 如果有人欺负您，是在哪段时间？ 1.最近1个月2.最近半年 3.最近1年 4.1年以前
19. 在您心情不好时，是否伤害过自己的身体？0.否 1.是

**儿童抑郁量表(CDI)**

| 指导语：根据你最近2周的实际感觉，请在最符合你情况的“口”内打“√”。**【备注：每个条目前加上“最近2周”】** | |
| --- | --- |
| 题号 | 选项 |
| 1 | 口我偶尔感到不高兴 口我经常感到不高兴 口我总是感到不高兴 |
| 2 | 口我不能解决任何问题 口我能解决部分问题 口我能解决任何问题 |
| 3 | 口我做任何事情都不会出错 口我做事情偶尔出错 口我做事情经常出错 |
| 4 | 口我做许多事情都有乐趣 口我做事情偶尔有乐趣 口我做任何事情都没有乐趣 |
| 5 | 口我的表现一直都像个坏孩子 口我的表现经常像个坏孩子 口我的表现偶尔像个坏孩子 |
| 6 | 口我偶尔担心不好的事情发生 口我经常担心不好的事情发生 口我总是担心不好的事情发生 |
| 7 | 口我恨我自己 口我不喜欢我自己 口我喜欢我自己 |
| 8 | 口所有不好的事情都是我的错 口许多不好的事情都是我的错 口仅有少数不好的事情是我的错 |
| 9 | 口我没有自杀的想法 口我想过去自杀但我不会去做 口我可能会自杀 |
| 10 | 口我每天都感觉想哭 口我经常感觉想哭 口我偶尔感觉想哭 |
| 11 | 口总是有事情干扰我 口经常有事情干扰我 口偶尔有事情干扰我 |
| 12 | 口我喜欢和别人在一起 口我经常不喜欢和别人在一起 口我总是不喜欢跟别人在一起 |
| 13 | 口我遇到事情总是拿不定主意 口我遇到事情经常拿不定主意 口我遇到事情很从容拿定主意 |
| 14 | 口我长得很好看 口我在长相上有些不如意 口我长得很丑 |
| 15 | 口我总是强迫自己去做作业 口我经常强迫自己去做作业 口我很容易完成作业 |
| 16 | 口我每天晚上很难睡着觉 口我经常晚上睡不着觉 口我睡觉很好 |
| 17 | 口我偶尔感到疲倦 口我经常感到疲倦 口我总是感到疲倦 |
| 18 | 口我总是感到不想吃东西 口我经常感到不想吃东西 口我胃口很好 |
| 19 | 口我不担心身体会疼痛 口我经常担心身体会疼痛 口我总是担心身体会疼痛 |
| 20 | 口我不感到孤独 口我经常感到孤独 口我总是感到孤独 |
| 21 | 口我总是感到上学无趣 口我偶尔感到上学有趣 口我经常感到上学有趣 |
| 22 | 口我有许多朋友 口我有一些朋友但我希望有更多朋友 口我没有任何朋友 |
| 23 | 口我在学校的学习还不错 口我的学习比以前稍差 口我以前功课很好现在很差 |
| 24 | 口我永远也不会像其他孩子那样棒 口如果我努力，我会像其他孩子那样棒 口我像其他孩子一样棒 |
| 25 | 口没有人真正地爱我 口我不确定是否有人爱我 口我确定有人爱我 |
| 26 | 口别人要我做的事，我通常会做 口别人要我做的事，我有时会做 口别人要我做的事，我从来不做 |
| 27 | 口我和别人相处很好 口我有时和别人发生矛盾 口我经常和别人发生矛盾 |

| **焦虑症状自评量表（GAD-7）【备注：每个条目前加“过去2周”】** | | | | | |
| --- | --- | --- | --- | --- | --- |
| 填写要求：在过去**的2周里，**评估是否存在下列描述的状况及频率，在符合的情形下选择打√ | | | | | |
| 序号 | **过去2周内的情形** | 情形发生频率及分值 | | | |
|  |  | 完全不会 | 好几天 | 超过一周 | 几乎每天 |
| 1 | 感觉紧张、焦虑或急切 | 0 | 1 | 2 | 3 |
| 2 | 不能够停止或控制担忧 | 0 | 1 | 2 | 3 |
| 3 | 对各种各样的事情担忧过多 | 0 | 1 | 2 | 3 |
| 4 | 很难放松下来 | 0 | 1 | 2 | 3 |
| 5 | 由于不安而无法静坐 | 0 | 1 | 2 | 3 |
| 6 | 变得容易烦恼或急躁 | 0 | 1 | 2 | 3 |
| 7 | 感到似乎将有可怕的事情发生而害怕 | 0 | 1 | 2 | 3 |
