# Supplementary material for: Impact of parental marital status on self-harm in Chinese primary school students: the mediating role of depression and the moderating effect of classmate relationships
Source: PeerJ. 2025 Apr 4;13:e19307. doi: 10.7717/peerj.19307 (PMC11974514; doi:10.7717/peerj.19307)
Supplement: Supplemental Information 3 [file peerj-13-19307-s003.doc]

Table 1. Structured questionnaire to collect sociodemographic information

1. School: ____
2. Grade: 1-6
3. Student ID：____
4. Sex: 1. Female 2. Male
5. Age:____
6. Place of household registration: 1. Urban 2. Rural
7. How is your relationship with your parents? 1. Good 2. General 3. Poor
8. What is the marital status of your parents? 1. Normal marriage 2. Separation（separated but not divorced） 3. Divorced
9. How would you describe your family's financial situation? 1. Good 2. General 3. Poor
10. Do you have any siblings? 0. No 1. Yes
11. Who mainly takes care of you? 1. Parents 2. Other relatives besides parents 3. Boarding at school 4. No one
12. How is your relationship with your parents? 1. Good 2. General 3. Poor
13. How are your relationships with your classmates at school? 1. Good 2. General 3. Poor
14. Do you feel a lot of pressure regarding your studies? 0. Low 1. General 2. High
15. How often do you use the internet or play with your phone? 1. Rarely use 2. Occasionally use 3. Frequently use
16. [Note: If the answer to question 4 is 1. Female, then show this question; otherwise, skip to question 17] Age at menarche ____ years old. (If you have not had menarche, please enter “0”) [Note: This question is limited to numeric responses, and the number cannot exceed your age (question 5)]
17. Have you been bullied at school? 1. No 2. Rarely 3. Several times 4. Often [Note: If you choose 1. No for this question, skip to question 19; otherwise, continue with question 18]
18. If you have been bullied, when did it happen? 1. In the last month 2. In the last six months 3. In the last year 4. More than a year ago
19. Have you ever hurt yourself when you were unhappy? 0. No 1. Yes

The Children's Depression Inventory (CDI)

****Instructions:**** Based on how you've been feeling over the past two weeks, please check the box that best describes your situation. [Note: Add "In the past two weeks" before each item.]

| **Item** | **Options** |
| --- | --- |
| 1 | ☐ I occasionally feel unhappy ☐ I often feel unhappy ☐ I always feel unhappy |
| 2 | ☐ I can't solve any problems ☐ I can solve some problems ☐ I can solve any problem |
| 3 | ☐ I never make mistakes in anything I do ☐ I occasionally make mistakes ☐ I often make mistakes |
| 4 | ☐ I enjoy many things ☐ I occasionally enjoy things ☐ I don't enjoy anything |
| 5 | ☐ I always behave like a bad child ☐ I often behave like a bad child ☐ I occasionally behave like a bad child |
| 6 | ☐ I occasionally worry about bad things happening ☐ I often worry about bad things happening ☐ I always worry about bad things happening |
| 7 | ☐ I hate myself ☐ I don't like myself ☐ I like myself |
| 8 | ☐ All bad things are my fault ☐ Many bad things are my fault ☐ Only a few bad things are my fault |
| 9 | ☐ I have no thoughts of killing myself ☐ I have thought about killing myself but I wouldn't do it ☐ I might kill myself |
| 10 | ☐ I feel like crying every day ☐ I often feel like crying ☐ I occasionally feel like crying |
| 11 | ☐ I'm always distracted ☐ I'm often distracted ☐ I'm occasionally distracted |
| 12 | ☐ I like being with others ☐ I often don't like being with others ☐ I always dislike being with others |
| 13 | ☐ I'm always indecisive ☐ I'm often indecisive ☐ I'm decisive |
| 14 | ☐ I look very good ☐ I'm somewhat unhappy with my appearance ☐ I look very ugly |
| 15 | ☐ I always force myself to do homework ☐ I often force myself to do homework ☐ I easily complete my homework |
| 16 | ☐ I have a hard time falling asleep every night ☐ I often have trouble sleeping ☐ I sleep well |
| 17 | ☐ I occasionally feel tired ☐ I often feel tired ☐ I always feel tired |
| 18 | ☐ I always feel like not eating ☐ I often feel like not eating ☐ I have a good appetite |
| 19 | ☐ I'm not worried about feeling pain ☐ I often worry about feeling pain ☐ I'm always worried about feeling pain |
| 20 | ☐ I don't feel lonely ☐ I often feel lonely ☐ I always feel lonely |
| 21 | ☐ I always find school boring ☐ I occasionally find school interesting ☐ I often find school interesting |
| 22 | ☐ I have many friends ☐ I have a few friends but wish I had more ☐ I have no friends |
| 23 | ☐ My schoolwork is okay ☐ My schoolwork has gotten a little worse than before ☐ My schoolwork used to be good but now it's bad |
| 24 | ☐ I'll never be as good as other kids ☐ If I try hard, I can be as good as other kids ☐ I'm as good as other kids |
| 25 | ☐ Nobody really loves me ☐ I'm not sure if anyone loves me ☐ I'm sure someone loves me |
| 26 | ☐ I usually do what others ask me to do ☐ I sometimes do what others ask me to do ☐ I never do what others ask me to do |
| 27 | ☐ I get along well with others ☐ I sometimes have conflicts with others ☐ I often have conflicts with others |

****Generalized Anxiety Disorder 7 (GAD-7) Scale****

****Instructions:**** Over the past two weeks, how often have you been bothered by any of the following problems? Please mark the appropriate box for each item.

| ****Item**** | ****Not At All**** | ****Several Days**** | ****More Than Half the Days**** | ****Nearly Every Day**** |
| --- | --- | --- | --- | --- |
| 1. Feeling nervous, anxious, or on edge | 0 | 1 | 2 | 3 |
| 2. Not being able to stop or control worrying | 0 | 1 | 2 | 3 |
| 3. Worrying too much about different things | 0 | 1 | 2 | 3 |
| 4. Trouble relaxing | 0 | 1 | 2 | 3 |
| 5. Being so restless that it is hard to sit still | 0 | 1 | 2 | 3 |
| 6. Becoming easily annoyed or irritable | 0 | 1 | 2 | 3 |
| 7. Feeling afraid as if something awful might happen | 0 | 1 | 2 | 3 |
