# Supplementary material for: Impact of parental marital status on self-harm in Chinese primary school students: the mediating role of depression and the moderating effect of classmate relationships
Source: PeerJ. 2025 Apr 4;13:e19307. doi: 10.7717/peerj.19307 (PMC11974514; doi:10.7717/peerj.19307)
Supplement: Supplemental Information 4 [file peerj-13-19307-s004.docx]

parent’smarriage1= Normal marriage

parent’smarriage2=Separation

parent’smarriage3= Divorced

classmaterelations1=Good

classmaterelations2=General

classmaterelations3=Poor
